# Effect of Heat Treatment Parameters on the Modification of Nano Residual Austenite of Low-Carbon Medium-Chromium Steel

**DOI:** 10.3390/nano13212829

**Published:** 2023-10-25

**Authors:** Yiran Wang, Ruian Wang, Wenzhen Yu, Yimin Gao

**Affiliations:** State Key Laboratory for Mechanical Behaviour of Materials, School of Materials Science and Engineering, Xi’an Jiaotong University, Xi’an 710049, China

**Keywords:** nano residual austenite, low-carbon medium-chromium steel, heat treatment, impact toughness

## Abstract

The ball milling lining board operates in a harsh environment, and the current materials fail to meet the requirements for large-sized boards due to the lack of synergistic properties between impact toughness and wear resistance. To address this issue, a low-carbon medium-chromium steel with martensite and nano residual austenite phases have been designed for future use. However, the residual austenite network could decrease the properties. Heat treatment, which includes processes like quenching and tempering, has the potential to alter the morphology and quantity of nano-scale residual austenite in the steel. In this study, the influence of heat treatment parameters on the morphologies and properties of steel has been investigated to address the wide-ranging fluctuations in impact toughness affected by nano residual austenite. Furthermore, the effect of cooling transformation on the microstructure has also been examined. The research findings indicate that modifying the quenching temperature of the steel within the range of 950–1100 °C results in a microstructure comprising martensite and nano residual austenite. At all quenching temperatures, the hardness exceeds 45 HRC, and the impact toughness shows a consistent improvement with increasing quenching temperature, indicating a modification of the nano residual austenite phase. The failure mode is primarily dimple fracture, with quasi-dissociation fracture as a secondary mode. The optimal heat treatment parameters are annealing at 930 °C, oil quenching at 1050 °C, and tempering at 250 °C. Under this condition, the steel exhibits a hardness of 51 HRC and impact toughness of 40 J/cm^2^ and an approximate fourfold increase compared to the untreated sample.

## 1. Introduction

The ball milling lining board is an essential metallic component in mining machinery known for its high wear resistance. It plays a crucial role in protecting the cylinder from impact failures caused by various types of media and materials. The environment in which the lining board operates is harsh, with statistics indicating an average consumption of about 0.2 kg of lining board per ton of mineral [1,2,3]. Currently, high-manganese steel, high-chromium cast iron, and medium- or high-carbon alloy steel are commonly used as metal lining boards worldwide [4]. Researchers have conducted studies to improve the performance and service life of these lining materials. For example, Petrico et al. [5] investigated the effects of casting process parameters on high-manganese steel ingots and found that higher Mn content increased the number and size of inclusions, while increasing C content favored inclusion formation. Consequently, high-manganese steel with higher ingot purity exhibited improved comprehensive performance. Erdakov et al. [6] focused on the formation characteristics of high-manganese steel plate structures and proposed a new production process that emphasized resource conservation. Mustafa et al. [7] studied the impact of B content on nickel-hard cast iron by adding boron-iron during the casting process. They found that higher B content increased carbide, hardness, and wear resistance but reduced toughness. Pavlina et al. [8] investigated the influence of adding Cu to medium-carbon steel on its hardenability. They found that lower concentrations of Cu improved the hardenability of the alloy steel. Even trace amounts of Cu can enhance the hardenability of medium-carbon steel, particularly when used as a lining board material. Guo et al. [9] explored the impact of adding Ti and RE elements to medium-carbon steel and their effects on the texture and properties of the steel. Their findings indicated that the secondary dendrite arm spacing tended to stabilize when the Ti content exceeded 0.066 wt.%, while the secondary dendrite arm spacing increased as the RE content reached 0.016 wt.%. The studies mentioned above have contributed to enhancing the wear resistance of current materials used for ball milling lining boards. However, their comprehensive properties still cannot meet the requirement of a longtime service life for the lining boards [10,11,12].

When high-manganese steel is used for ball mill lining boards, its work hardening characteristics lead to a noticeable reduction in wear resistance, as the subsurface hardness after wear is only in the range of 240–350 HBW [13]. Additionally, the material’s low yield strength makes it susceptible to deformation during service. High chromium cast iron, on the other hand, boasts excellent wear resistance. However, it faces challenges in achieving both toughness and wear resistance simultaneously, limiting its application to smaller lining boards [14]. Medium- and high-carbon alloy steels, featuring Cr-Mo or Cr-Ni-Mo systems with a carbon content ranging from 0.5 wt.% to 1.0 wt.%, demonstrate good toughness and wear resistance. These steels are suitable for various sizes of lining boards. However, it has been observed that large diameter lining boards (≥6 m) made from such steel still exhibit insufficient toughness [15,16]. To address these limitations and improve the performance of ball mill lining boards, ongoing research and development efforts are focused on optimizing the composition and production processes of these materials.

To enhance the toughness of large lining boards, a low-carbon medium-chromium steel has been developed. This steel is designed with a controlled carbon content of 0.20–0.25 wt.% and is alloyed with chromium, nickel, molybdenum, and other elements. The microstructure of the steel contains martensite and residual austenite phases. The reason for the existence of the residual austenite is that the martensite transformation has not been complete. However, residual austenite network decreases the mechanical and anti-wear resistance properties. The heat treatment process, encompassing annealing, quenching, and tempering, has the potential to alter the morphology and quantity of nano-scale residual austenite in the steel, and then improve its overall toughness and wear resistance. Achieving the desired combination of high impact toughness and sufficient hardness is a complex task, requiring a systematic approach to explore its heat treatment parameters. Unfortunately, there has been limited research conducted on the heat treatment process for this material, particularly regarding the influence of holding temperature on its microstructure and properties.

In this study, the influence of heat treatment processes on the modification of nano residual austenite of low-carbon medium-chromium steel is investigated. The effect of cooling transformation on the microstructure is also examined. The quenching and tempering parameters of the steel are studied to identify key factors affecting its impact toughness by modify nano residual austenite. These findings aim to provide a foundation for industrialization and consistent application of ball milling lining boards.

## 2. Experiments

### 2.1. Materials

Medium-frequency induction furnaces are employed to smelt the low-carbon medium-chromium steel through the sand-casting process. During the process, the melting temperature is sustained at 1550~1600 °C. The designed chemical composition and the measured composition of the materials are detailed in Table 1.

The sample is cast into a Y-shaped sample, and its metallographic observation is conducted. The samples undergo heat treatment process including annealing, quenching, and tempering with different parameters, and Table 2 displays the number of heat treatment samples.

### 2.2. Microstructure Investigation and Properties Test

The microstructure analysis of the low-carbon medium-chromium steel is conducted using scanning electron microscopy (SEM). Additionally, phase analysis is performed using an XRD detector (PANalytical X’PERTMPD, Malvern, Cambridge, UK) with an angular 2θ range of 20° to 90° and a step size of 0.02°.

The continuously cooling transformation (CCT) curve is utilized as a crucial reference for analyzing and predicting the austenitic transformation, transformation products, and properties of the steel during continuous cooling [17]. It is critical for modifying the nano residual austenite. The CCT curve is derived from thermodynamic calculations and, although it may have some inaccuracies due to theoretical limitations, its findings are supplemented and verified through further dynamic studies. The temperature expansion curve of the steel is obtained using the Gleeble3500 thermal simulation tester (Poestenkill, NY, USA). The process involves a heating stage at a rate of 10 °C/s from 25 °C to 600 °C, followed by 0.05 °C/s between 600 °C and 1100 °C. The expansion curve depicts the elongation of the size and linear temperature change initially due to thermal expansion, which continues to increase with temperature. Beyond a specific critical temperature, there is a downward inflection point caused by volume contraction due to a new phase transition [18]. Further heating to the full extent of austenite transformation leads to an increase in the size of the steel due to thermal expansion. The Gleeble3500 thermal simulation tester was used to measure the results, and the tangent method was employed to determine the phase transformation temperatures.

Vickers hardness is measured in this research, and the values are obtained directly from the software after the indentation. Each specimen is tested 10 times, and the average value is calculated to ensure the accuracy and reliability of the test data. The loading force for the hardness test is 10 gf (0.098 N), and the holding time is 10 s.

**Figure 1:**
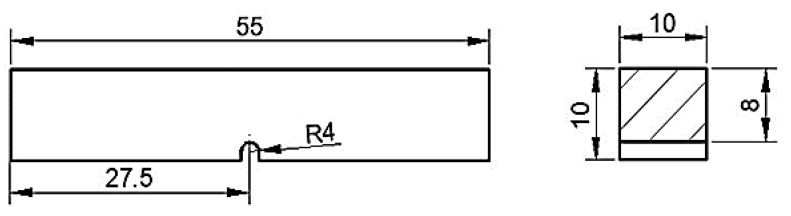
Schematic of impact toughness test sample.

The impact toughness test is conducted using the JBW-300 testing machine (Nanjing, China). The sample is made with a U-notch (as shown in Figure 1), and the surface is polished and ultrasonically cleaned for 15 min to achieve a roughness (Ra) of ≤5.0 μm. The standard sample is then placed on the testing machine, and the impact test is carried out with a pendulum with a measuring range of 150 J.

A pin-on-disk two body wear test is conducted to evaluate the wear resistance of the steel [19]. The counterpart material is SiC ceramic garnet paper. The size of the samples is 10 mm × 10 mm × 10 mm with flat ended. The test parameter is shown in Table 3. All wear tests are conducted 3 times under identical conditions. The entire samples are weighed before and after the wear experiments. Then, the relation Formula (1) is used to calculate the wear rate.

(1) $$\Delta W=\frac{\Delta W_{1}+\Delta W_{2}+\Delta W_{3}}{3}$$

where Δ*W*_1_ is the weight loss of the sample after the 1st test, Δ*W*_2_ is the weight loss of the sample after the 2nd test, Δ*W*_3_ is the weight loss of the sample after the 3rd test, Δ*W* is the average value of Δ*W*_1_, Δ*W*_2_, Δ*W*_3_ is the weight loss of wear.

## 3. Results and Discussion

### 3.1. As-Cast

**Figure 2:**
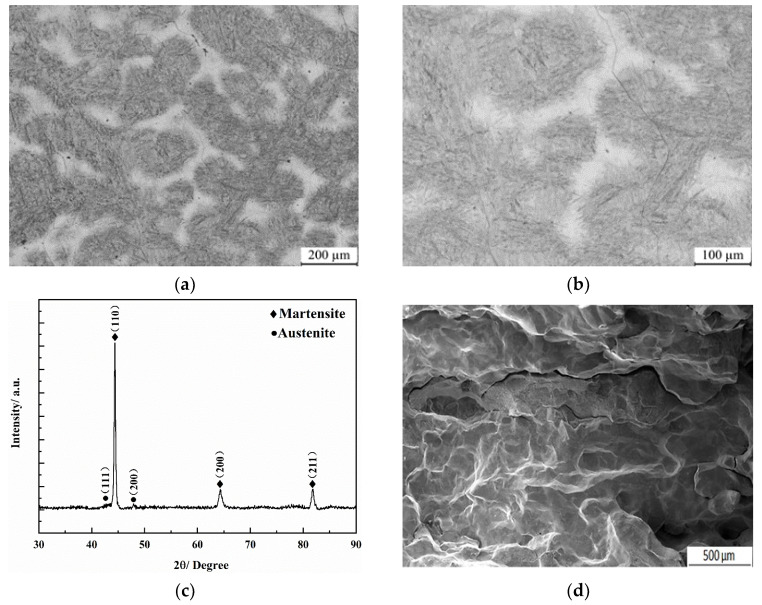
As-cast state morphology and XRD pattern of low-carbon medium-chromium steel. (**a**) Residual austenite at low magnification. (**b**) Residual austenite at high magnification. (**c**) XRD pattern. (**d**) Impact fracture morphology.

Figure 2a,b shows the as-cast morphologies of the designed low-carbon medium-chromium steel. The black area is martensite, whereas the white area is the residual austenite phase based on the composition characteristics of the steel. The residual austenite in the as-cast state exhibits non-continues network and agglomerate as micro scale. In this case, the microhardness measures 720 HV in the black area and 637 HV in the white area.

Figure 2c shows the XRD pattern of the as-cast state, indicating the presence of martensite and austenite diffraction peaks. This suggests that the microstructure primarily consists of a martensite matrix. According to the austenite transformation rule of the steel, there is an expectation of achieving nano residual austenite in the microstructure. The Rockwell hardness test score for the steel is 50 HRC, indicating a high level of hardness. However, the impact toughness test conducted on the as-cast sample yielded a value as low as 8.1 J/cm^2^. Figure 2d presents the SEM morphology of the impact fracture, displaying a polygonal quality characteristic of intergranular brittle fracture. Based on the insufficient impact toughness value and the brittle intergranular fracture displayed by the as-cast steel sample, it does not meet the necessary criterion (at least 30 J/cm^2^) for use as a material for large ball milling lining boards. To enhance the impact toughness of the steel, appropriate heat treatment parameters can be employed. These processes can help modify the nano-scale residual austenite and enhancing its uniformity, and increase the toughness of the steel, making it more suitable for applications that require higher impact resistance.

Although the as-cast microstructure of the steel is primarily martensite, the faster cooling rate during the casting process leads to component segregation, and the SEM images also indicate poor uniformity in the microstructure and a low property. Consequently, it is not feasible to directly utilize the cast sample, which must undergo appropriate heat treatment processes to modify the retained austenite as nano scale and enhance the performance of the steel.

### 3.2. Cooling Transformation Curve and Critical Cooling Rate

**Figure 3:**
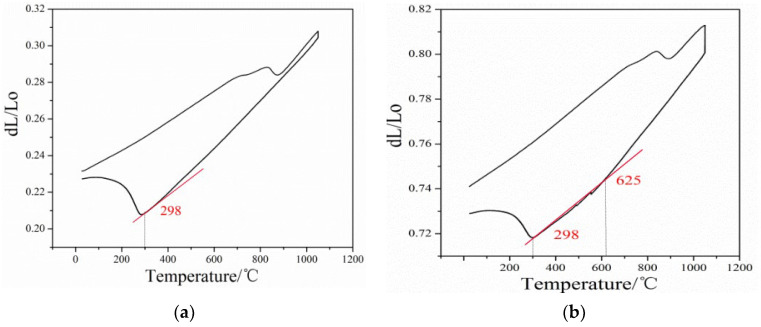
Relationship between volume variation and different cooling rates of the steel: (**a**) 0.1 °C/s, (**b**) 0.05 °C/s.

Figure 3 illustrates the relationship between volume variation and different cooling rates for the steel. At a cooling rate of 0.1 °C/s, the phase transformation process shows only one martensite transformation occurring at 298 °C. However, when the cooling rate is reduced to 0.05 °C/s, two phase transitions are observed. The first transition is the pearlite transformation, which takes place at 625 °C. The second transition is the martensitic transformation, occurring at 298 °C.

**Figure 4:**
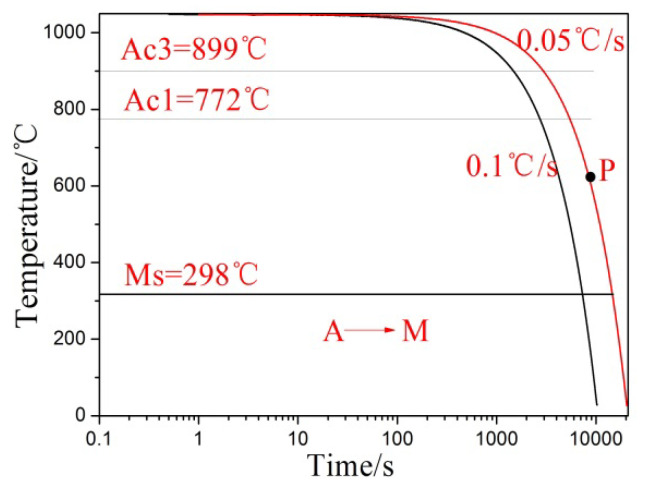
Cooling rates and continuous cooling transition curves.

Based on the provided curves, the phase transition temperatures for martensite and pearlite are determined at different cooling rates for the steel. Additionally, Figure 4 displays the continuous cooling transition curves of the steel, demonstrating that the phase transformation pattern changes with varying cooling rates. The steel’s phase transformation temperature is determined using the tangent method [20], with results indicating Ac1 = 772 °C and Ac3 = 899 °C for the designed low-carbon medium-chromium steel. The composition of the steel includes multiple alloying elements, which contribute to the relative stability of austenite and inhibit the transformation into pearlite. However, when the steel is cooled at a slower rate of 0.05 °C/s, the formation of pearlite structure is observed. This indicates that the cooling rate plays a significant role in the phase transformation behavior of the steel.

Based on the above discussion, the cooling transformation curve of the steel indicates that the critical cooling rate is relatively low. This is attributed to the pearlite transition in the austenite, which belongs to a diffusion-type phase transition [21]. The addition of a significant number of alloying elements, particularly Mo, enhances the stability of austenite. When these alloying elements dissolve into the austenite, the isothermal transition diagram shifts to the right, delaying the pearlite transition, and reducing the critical quenching rate of the steel. As a result, the Ms point of martensitic transformation is also reduced.

**Figure 5:**
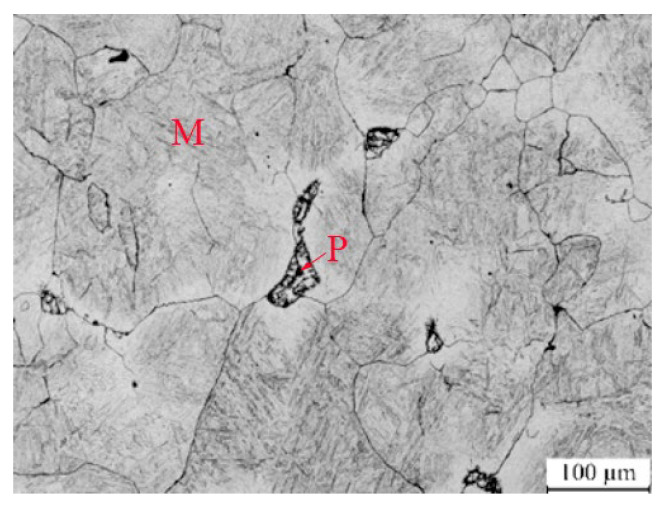
The microstructure of the designed low-carbon medium-chromium steel at a cooling rate of 0.05 °C/s (M: martensite; P: pearlite).

To examine the microstructure of the designed low-carbon medium-chromium steel at a cooling rate of 0.05 °C/s, SEM was employed, and the results are presented in Figure 5. The SEM images illustrate that the microstructure of the steel primarily consists of martensite with only a small amount of pearlite. This suggests that the critical cooling rate of the steel is low, indicating strong hardenability and facilitating the attainment of martensitic and nano residual austenite. These observations align with the theoretical calculation results.

### 3.3. Annealing Process

**Figure 6:**
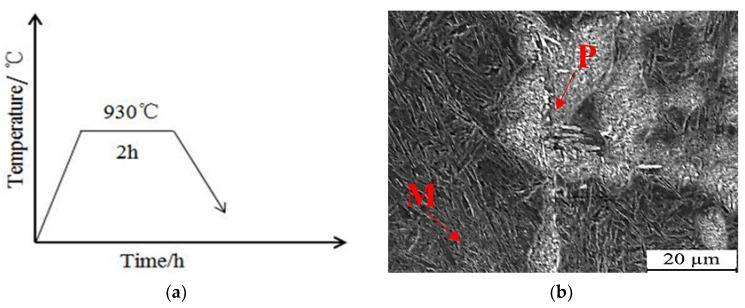
Annealing process and microstructure of low-carbon medium-chromium steel. (**a**) Annealing process. (**b**) SEM morphology. (M: martensite; P: pearlite).

Figure 6a depicts the annealing process for the steel. The steel investigated in this study was produced through the casting process. However, the as-cast state often results in faster cooling rates, which can lead to component segregation and affect the uniformity of both the microstructure and mechanical properties. To address these issues and enhance the uniformity and properties of the steel, a complete annealing process was employed. For the annealing process, the steel was heated to a temperature 30–50 °C above Ac3, which is the temperature at which phase transition occurs. In this particular study, the annealing temperature applied was determined to be 930 °C based on the known phase transition temperature. Once this set temperature is reached, the steel is cooled down along with the furnace. When the temperature drops to approximately 400 °C, the sample is then removed for air cooling. To determine the appropriate holding time for the annealing process, it is necessary to refer to relevant literature and consider the thickness of the samples. The relationship between holding time and sample thickness is described by Formula (2), as stated in the literature [22]:

*τ* = α·*K*·*D* (2)

where: *τ* is the holding time/min; α is heating coefficient/min·mm^−1^; *K* is the correction coefficient of sample loading mode; and *D* is the effective thickness/mm of the sample.

Using the above formula where α = 2 min/mm^−1^, *K* = 3, and *D* = 20 mm, the holding time for annealing of the steel is calculated as: *τ* = 2 × 3 × 20 min = 2 h. Complete annealing is employed to eliminate component segregation in the steel casting process, ensuring a uniform composition and improved performance. SEM morphology of the steel after annealing at 930 °C is shown in Figure 6b, revealing a microstructure consisting of martensite and pearlite. The presence of a small amount of carbon and higher chromium content in the steel enhances its hardenability, allowing it to achieve a martensitic structure even with slow annealing cooling rates in the furnace.

Following the annealing process at 930 °C, mechanical properties testing was conducted on the steel. The results of these tests are presented in Table 4. When comparing the mechanical properties of the annealed steel samples, it can be observed that the microstructure contains martensitic and pearlite phases at room temperature. As a result of the annealing process, the hardness of the steel decreases while the impact toughness remains relatively unchanged.

### 3.4. Quenching Process

#### 3.4.1. Quenching Temperature

The low-carbon medium-chromium steel used in this experiment is hypoeutectoid and typically requires heating to a temperature 30–50 °C above Ac3. Heating below Ac3 results in the formation of not only martensite but also nano residual austenite phases in the microstructure, leading to a decrease in the hardness and strength of the steel. On the other hand, if the quenching temperature is higher than Ac3, the austenite grains may coarsen. Due to the presence of various alloying elements, the dissolution and redistribution of this particular steel require more energy compared to regular carbon steel. Additionally, some elements, especially those that form strong carbides, have higher diffusion activation energies in the austenite phase, and coarsening occurs at higher temperatures. To expedite the austenitizing process, a higher quenching temperature above Ac3, specifically at 50 °C intervals, is employed. The quenching temperatures of 950 °C, 1000 °C, 1050 °C, and 1100 °C are set in order to investigate the impact of different quenching temperatures on the microstructure and properties of the steel. Once the quenching temperature is determined, the next step is to determine the holding time. The holding time is crucial as it affects the solubility of carbides in the steel, which in turn influences the degree of austenite transformation and grain size. Insufficient holding time can lead to incomplete austenitization, while excessive holding time can result in coarse austenite grains. Therefore, when performing the quenching process, both the heating temperature and holding time must be carefully considered. The calculation for the holding time during steel austenitization can be determined using Formula (3) as provided in the literature [23]. This formula helps to determine the optimal holding time required for achieving complete austenitization of the steel.

*τ* = *K*·*D* (3)

where: *τ* is the holding time/min; *K* is the heating coefficient/min·mm^−1^; *D* is the finite thickness of the sample/mm.

In the formula above, with *K* = 2 min·mm^−1^ and *D* = 20 mm, the holding time for quenching is obtained as *τ* = 2 × 20 min = 40 min. The quenching medium, typically consisting of water, saltwater, or oil, is commonly employed to cool the steel from its austenitic state to a temperature below the Ms point. In order to obtain the martensitic structure from the steel after quenching, the cooling rate during quenching must be higher than the critical cooling rate. Previous studies showed that the critical cooling rate of the steel is less than 0.1 °C/s, which indicates that the martensitic structure can be obtained even after relatively slow cooling during quenching. Therefore, three quenching modes, including water, air, and oil cooling, are set to explore the influence of different quenching media on the microstructure and properties of the steel in this study.

**Figure 7:**
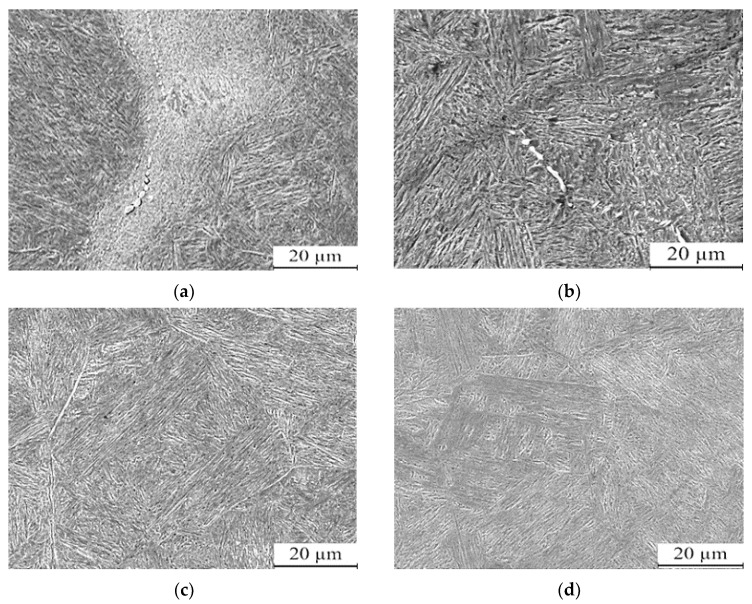
Microstructure of low-carbon medium-chromium steel subjected to various quenching temperatures: (**a**) 950 °C, (**b**) 1000 °C, (**c**) 1050 °C, (**d**) 1100 °C.

Figure 7 illustrates the microstructure of the steel after undergoing different quenching temperatures. The gray phase corresponds to martensite, while the white nano phase is indicative of residual austenite. The heat treatment process results in a transformation of the steel’s microstructure to tempered martensite and nano residual austenite, as shown in the figure. The specific quenching temperature has an impact on the resulting microstructure, leading to variations in the distribution of martensite and residual austenite within the steel.

As the quenching temperature increases, the size and spacing of the martensitic laths in the microstructure gradually increase. At temperatures of 950 °C and 1000 °C, irregularly arranged white nano residual austenite can be observed at the grain boundaries.

**Figure 8:**
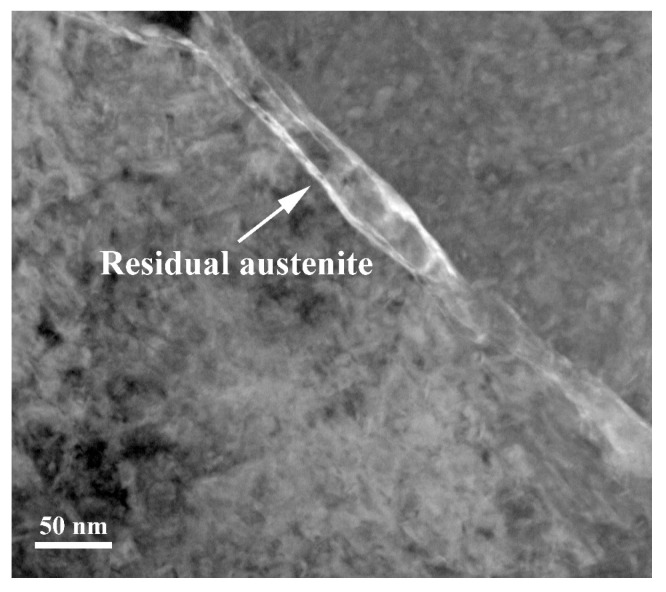
TEM morphologies of low-carbon medium-chromium steel at 1050 °C quenching temperatures.

At a quenching temperature of 1050 °C, the martensite and nano residual austenite phases become more uniform, and the grain size becomes finer. However, when the quenching temperature reaches 1100 °C, nano residual austenite is same as 1050 °C but the martensite laths become wider, and the grain size becomes relatively coarse. The TEM observation has been conducted to prove the characterization of nano residual austenite as shown in Figure 8. From the TEM morphologies, it can be inferred that the size of residual austenite decreases to 50~100 nm.

This phenomenon can be attributed to the interconnected relationship among the quenching temperature, grain size, and the dissolution of alloy elements in austenite. When the quenching temperature increases, alloy elements have a greater tendency to solidify into the austenite, thereby increasing its stability. This, in turn, affects the hardenability of the steel and the formation of lath martensite.

**Figure 9:**
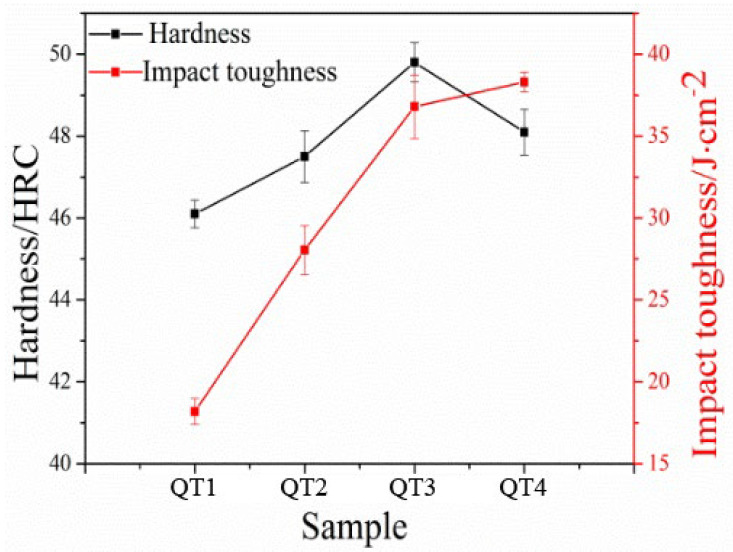
Hardness and impact toughness of low-carbon medium-chromium steel at various quenching temperatures.

This study conducted hardness and impact toughness tests on QT1–QT4 samples at various quenching temperatures. As shown in Figure 9, the macro hardness of all samples is consistently above 45 HRC across the different quenching temperatures (950–1100 °C). Initially, as the quenching temperature increases, the hardness also increases, reaching a peak value of 49.8 HRC at 1050 °C. However, at 1100 °C, the hardness starts to decrease. The reason behind this trend is that as the quenching temperature rises, the process of austenitization becomes more complete, and the diffusion driving force of alloying elements increases. This leads to the precipitation of alloying elements within the austenite, enhancing the hardenability of the steel and resulting in the formation of more martensitic structures. At 1050 °C, the resulting martensitic and nano residual austenite are uniform with a relatively fine grain size, which contributes to higher hardness. However, as the quenching temperature further increases, the austenite grains grow larger, causing the final martensitic structure to become coarser. According to the empirical formulas [24], it is known that the size of grain and lath decreases with decreasing austenite grain size.

As the quenching temperature increases, there is a gradual improvement in impact toughness, although the increase becomes relatively slight after reaching 1050 °C. This can be attributed to several factors. Firstly, different quenching temperatures result in varying degrees of austenitization and content of alloying elements dissolved into the austenite. These factors influence the toughness of the steel [25]. Additionally, the nano residual austenite and grain size play significant roles in determining the steel’s toughness. When the quenching temperature is too high, the grain size becomes relatively coarse, reducing the effectiveness of grain boundaries and making the steel more prone to deformation under external pressure.

**Figure 10:**
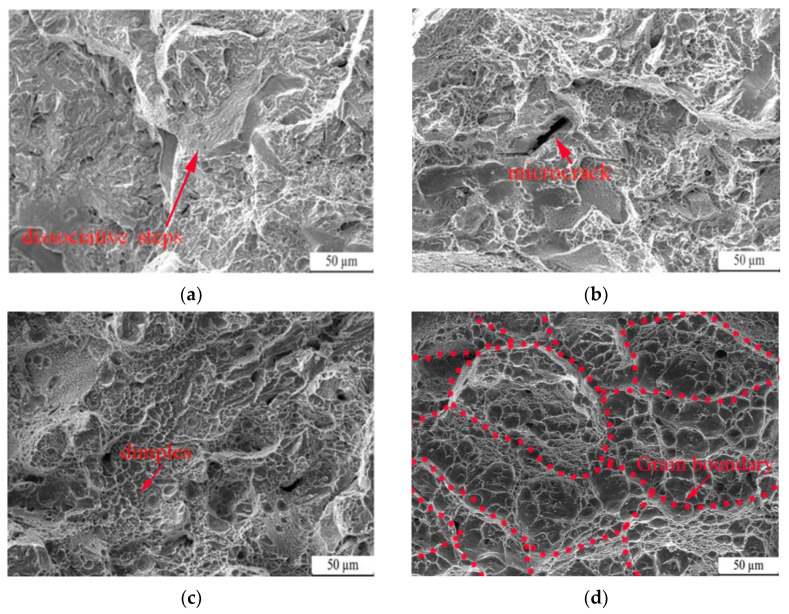
Fracture morphology of low-carbon medium-chromium steel at various quenching temperatures: (**a**) 950 °C, (**b**) 1000 °C, (**c**) 1050 °C, (**d**) 1100 °C.

Figure 10 depicts the fracture morphologies of QT1–QT4 samples subjected to different quenching temperatures. These fracture images exhibit a typical dimple structure, with varying quantities and depths of dimples for samples tempered at different temperatures. The primary failure mode is a dimple fracture, with a quasi-dissociation fracture acting as a secondary mode. At a quenching temperature of 950 °C, the fracture appears flat with a dissociation surface, and only a few shallow dimples, resulting in low impact toughness. When quenched at 1000 °C, visible cracks are present in the fracture, indicating poor toughness. However, at 1050 °C, there is an increase in the number and density of dimples, leading to improved toughness. Finally, at 1100 °C, densely packed dimples are observed, indicating typical ductile fracture behavior, which is indicative of higher toughness.

#### 3.4.2. Quenching Media

**Figure 11:**
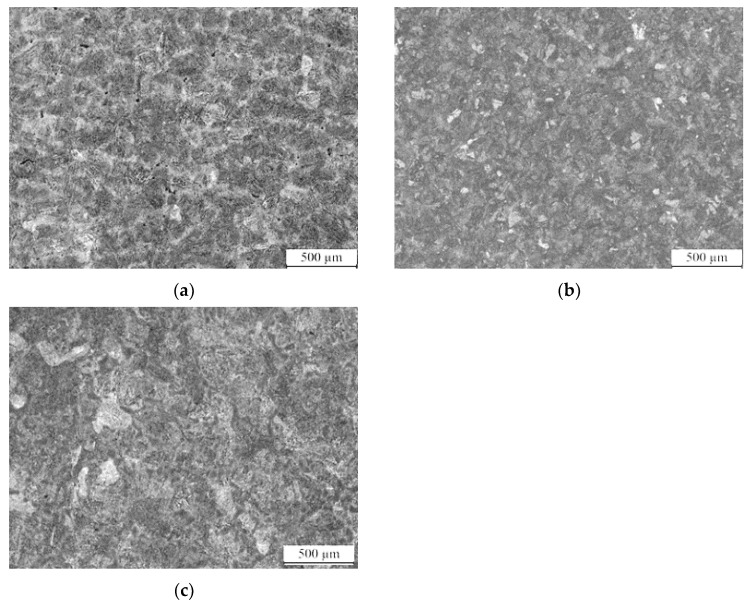
Microstructure of low-carbon medium-chromium steel under different quenching media. (**a**) Water. (**b**) Oil. (**c**) Air.

Figure 11 presents the microstructure of the designed steel under different quenching media. The image reveals that the steel obtained the martensite and nano residual austenite phases regardless of the cooling method employed. The high hardenability of the steel is attributed to the addition of a significant number of alloying elements, which inhibits the decomposition of supercooled austenite. The cooling rates of all three media are well above the critical cooling rate, resulting in the formation of martensite and nano residual austenite at room temperature. However, there are variations in the amount and uniformity of nano residual austenite in the microstructure based on the quenching media. When oil is used as the quenching medium, the microstructure exhibits good organizational uniformity and relatively small-sized nano residual austenite. When using water and air cooling, relatively larger nano-sized residual austenite is observed, along with discontinuous martensitic laths. This discrepancy is due to the faster cooling rate of water, which provides sufficient driving force for the rapid martensitic transformation within a shorter transformation time. While the number of martensitic laths and the size of the nano residual austenite increases, the uniformity of the microstructure becomes poorer.

**Figure 12:**
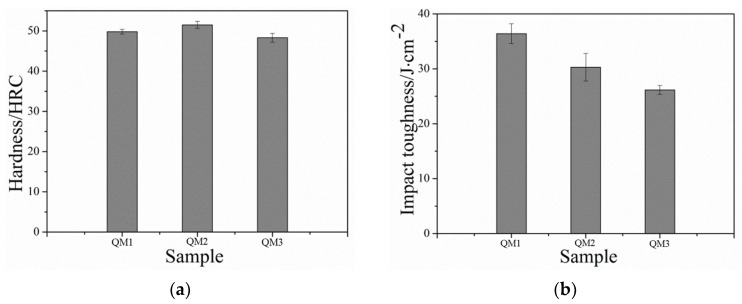
Hardness and impact toughness of low-carbon medium-chromium steel under different quenching media. (**a**) Hardness (**b**) Impact toughness.

Figure 12a illustrates the hardness results of the QM1, QM2, and QM3 samples after being quenched with different media. The data indicates that the differences in hardness among the steels quenched with different media at the same quenching temperature are relatively small. Overall, the oil-quenched sample (QM2) exhibits the highest hardness, followed by the water-quenched sample (QM1), while the air-quenched sample (QM3) demonstrates the lowest hardness. This trend can be explained by considering the cooling rates of the quenching media. Although the critical cooling rate for martensite formation in the steel is relatively low, all three media exceed this critical cooling rate. However, due to the faster cooling rate of water as a quenching medium, the transformation of austenite occurs at a faster rate, thereby increasing the probability of martensite nucleation. As a result, the size of residual austenite in the microstructure becomes smaller and more uniformly, leading to the highest hardness observed in the water-quenched sample. Conversely, the slower cooling rate of air quenching results in reduced martensite formation and a bigger residual austenite, leading to a lower hardness in the air-quenched sample.

**Figure 13:**
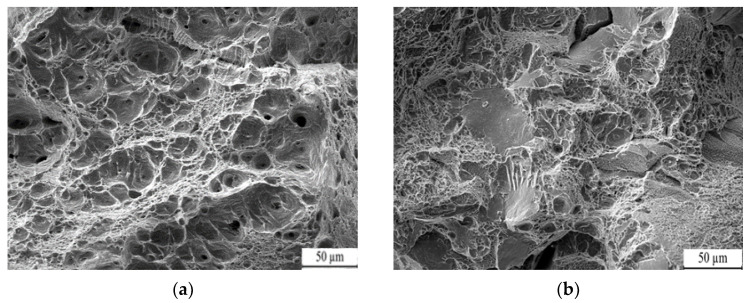
Fracture morphology of low-carbon medium-chromium steel at various quenching media. (**a**) Oil. (**b**) Air.

Figure 12b demonstrates the impact toughness test results of the QM1, QM2, and QM3 samples quenched with different media. The water-quenched sample (QM1) exhibited the highest impact toughness at the same quenching temperature, followed by the oil-quenched sample (QM2), while the air-cooled sample (QM3) displayed the lowest impact toughness. The superior impact toughness of the water-quenched sample can be attributed to the fast cooling and strong quenching capacity of water, which subjects the steel to greater stress. Additionally, the cooling characteristics of water are less efficient, resulting in a varying cooling rate in different temperature intervals. Specifically, the cooling rate during the martensitic transition temperature interval, which requires slower cooling, is excessively high in water quenching. Conversely, the cooling rate of oil is lower in the temperature range of 200–300 °C, leading to appropriate nano residual austenite after oil quenching. The microstructure resulting from air cooling is irregular, contributing to comparatively lower toughness. Furthermore, Figure 13 illustrates the impact fractures of the samples subjected to different quenching media. The oil-quenched sample (QM2) exhibits densely distributed and abundant dimples, indicating a high level of impact toughness. In contrast, the water-quenched sample (QM1), as in Figure 10c, shows visible cracks and tearing edges, resulting in reduced impact toughness. The air-cooled sample (QM3) displays inadequate dissociation surfaces, cracks, and dimples, suggesting a mixed fracture mode of quasi-dissociation and dimple fracture, leading to lower impact toughness. In summary, different quenching media produce dimples in the impact fractures of the steel, with ductile fracture being the primary mode.

### 3.5. Tempering Process

**Figure 14:**
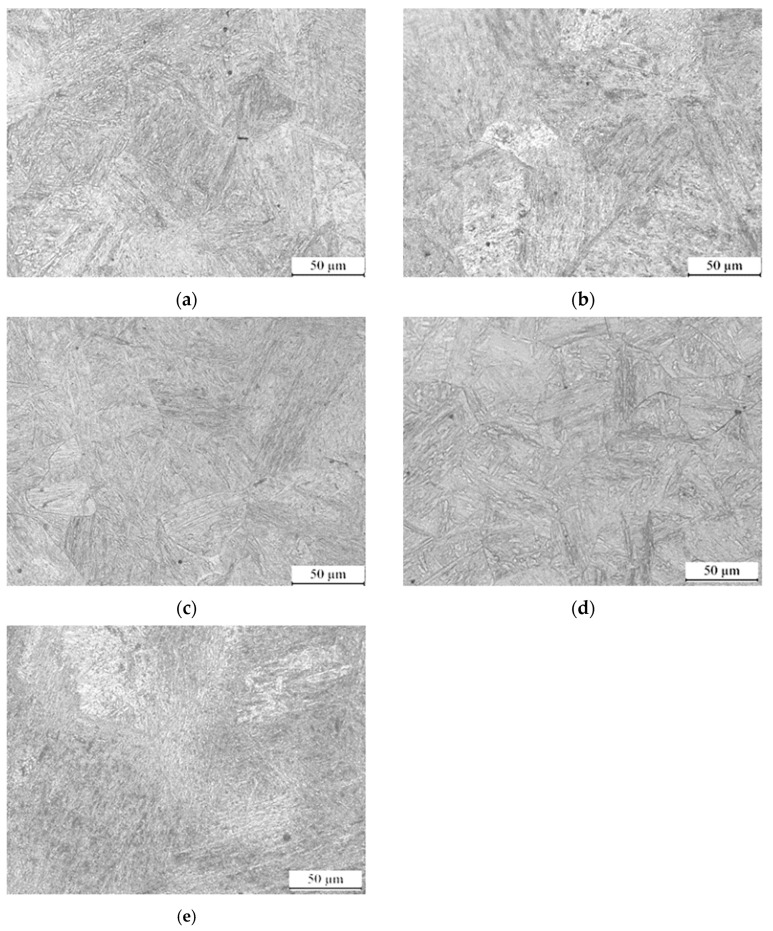
Microstructure of low-carbon medium-chromium steel at various tempering temperatures: (**a**) 200 °C, (**b**) 250 °C, (**c**) 300 °C, (**d**) 350 °C, (**e**) 400.

The influence of tempering temperature on the microstructure of low-carbon medium-chromium steel is investigated, as depicted in Figure 14. The microstructure of the steel after tempering at various temperatures is analyzed. At tempering temperatures of 200–250 °C, the martensite and nano residual austenite phases distribution is uniform. The tempered steel exhibits super fine-grained martensite and nano residual austenite with good mechanical properties. Tempering at 300 °C leads to grain growth and coarsening of the martensitic lath structure. This results in a slightly larger grain size compared to the previous tempering temperature range, which may have a marginal effect on the mechanical properties of the steel. When tempered at 350 °C, the microstructure exhibits poor uniformity with coarser grains. The larger size nano residual austenite may impact the mechanical properties, potentially reducing the strength and toughness of the steel. At a tempering temperature of 400 °C, martensite decomposes, and recrystallization of the ferrite phase has not been completely accomplished at this stage. Overall, the martensite and nano residual austenite of the steel are significantly affected by the tempering temperature. It is important to select an appropriate tempering temperature to achieve the desired mechanical properties and microstructural characteristics for specific applications.

**Figure 15:**
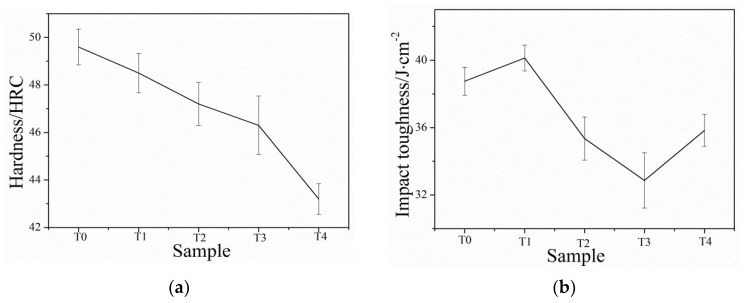
Hardness and impact toughness of low-carbon medium-chromium steel at various tempering temperatures. (**a**) Hardness. (**b**) Impact toughness.

Figure 15a presents the results of hardness testing conducted at various tempering temperatures on the low-carbon medium-chromium steel. The hardness values are shown to decrease as the tempering temperature increases. At lower tempering temperatures, the alloy elements within the steel dissolve into the martensite phase, resulting in a higher hardness. This is because the dissolved alloy elements strengthen the martensite structure. The strengthening effect is due to the solute atoms impeding dislocation motion, thereby increasing the resistance to deformation. However, as the tempering temperature increases, the carbon content within the martensite phase decreases. This carbon loss occurs due to diffusion of carbon atoms and facilitated by the elevated temperature during tempering. Additionally, the growth in nano residual austenite further consumes the carbon content within the martensite structure. With the decrease in carbon content and the growth in nano residual austenite, the overall microstructure undergoes changes. Specifically, the martensitic lath structure coarsens, resulting in larger grain sizes. This coarsening effect occurs due to the reduction in carbon content and the growth of nano residual austenite, which act as nucleation sites for grain growth. These changes in the microstructure, including the reduction in carbon content and the coarsening of grain sizes, contribute to the decrease in hardness observed at higher tempering temperatures. Moreover, at a tempering temperature of 400 °C, a significant portion of the martensite phase decomposes, and nano residual austenite continues to grow. This further diminishes the hardness of the steel. In summary, the evolution of hardness with tempering temperature reflects the changes in the microstructure, including the reduction in carbon content, the growth in nano residual austenite, and the coarsening of grain sizes. Understanding the relationship between tempering temperature and hardness is crucial for tailoring the mechanical properties of low-carbon medium-chromium steel for specific applications.

Figure 15b illustrates the impact toughness testing results of T0–T4 samples at different tempering temperatures. The figure indicates that the impact toughness initially remains relatively stable within a narrow range of tempering temperatures. However, a gradual decrease in impact toughness is observed with a further increase in tempering temperature. Within the range of 200 °C to 250 °C, the impact toughness of the steel shows signs of recovery. This recovery is attributed to several factors [26]. Firstly, the martensite undergoes recrystallization, leading to the formation of a more favorable microstructure. Secondly, the nano residual austenite in the microstructure begins to refine, resulting in a reduction in stress concentration points. Lastly, the dislocation slip within the martensite structure is hindered, contributing to increased toughness. On the other hand, when the tempering temperature is within 300 °C to 400 °C, the martensite in the microstructure undergoes decomposition making them prone to crack initiation and growth. As a result, the steel exhibits lower impact toughness, a phenomenon known as low-temperature tempering brittleness. Interestingly, the impact toughness of the T5 sample at a tempering temperature of 400 °C shows improvement. This improvement can be attributed to the further enhancement of the steel’s toughness and the avoidance of low-temperature tempering brittleness.

**Figure 16:**
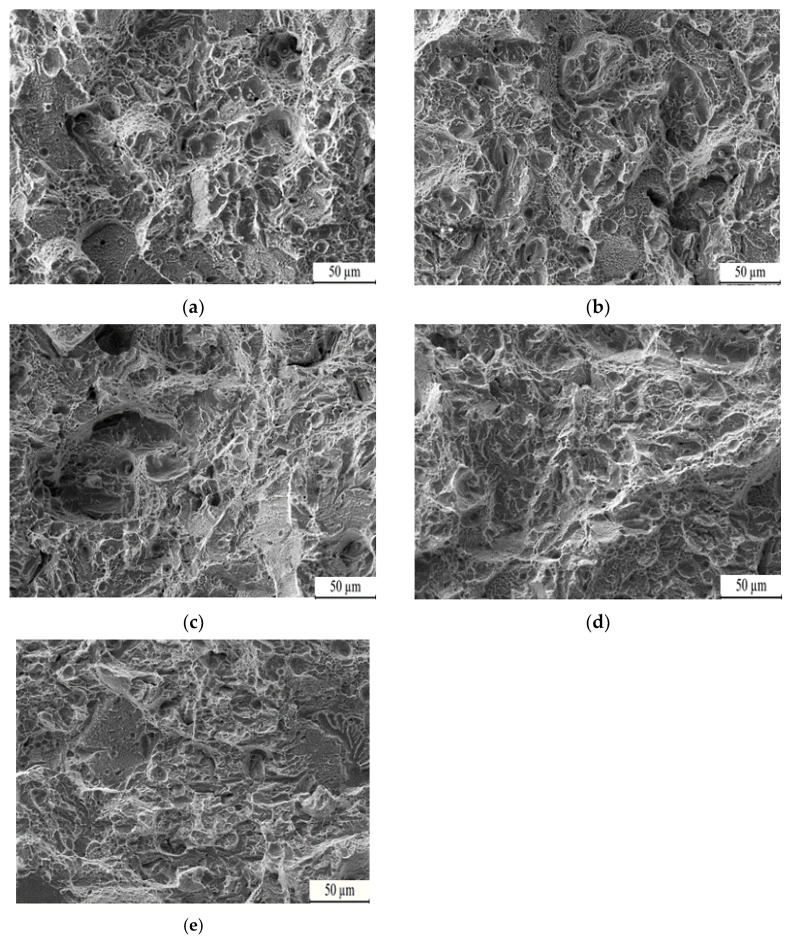
Fracture morphology of low-carbon medium-chromium steel at various tempering temperatures: (**a**) 200 °C, (**b**) 250 °C, (**c**) 300 °C, (**d**) 350 °C, (**e**) 400.

Analyzing the impact fracture of the T0–T4 samples at different tempering temperatures, as shown in Figure 16, reveals specific features. After tempering, the impact fractures exhibit the presence of dimples at various temperatures. This indicates that the main fracture mode is ductile fracture after tempering. Tempering temperatures within the range of 200 °C to 250 °C results in a significant number of dimples on the fracture surface. However, at tempering temperatures of 300 °C to 350 °C, the fracture surface shows tearing edges, detached planes, and even a small number of holes. Additionally, the grain size of the samples becomes larger, leading to reduced toughness. At a tempering temperature of 400 °C, the number of dimples increases. Furthermore, the dimples are deep and densely distributed, forming a microporous aggregation on the fracture surface.

The microstructure obtained after annealing at 930 °C, oil quenching at 1050 °C, and tempering at 250 °C primarily consists of martensite and fine nano residual austenite. This combination of microstructures contributes to well-balanced properties in the steel, with an impact toughness of 40 J/cm^2^. This value is much higher than the reported results [5,6,7,8,9,10,11,12] and shows a great application prospect for use in the ball milling lining board.

### 3.6. Wear Test Results

**Figure 17:**
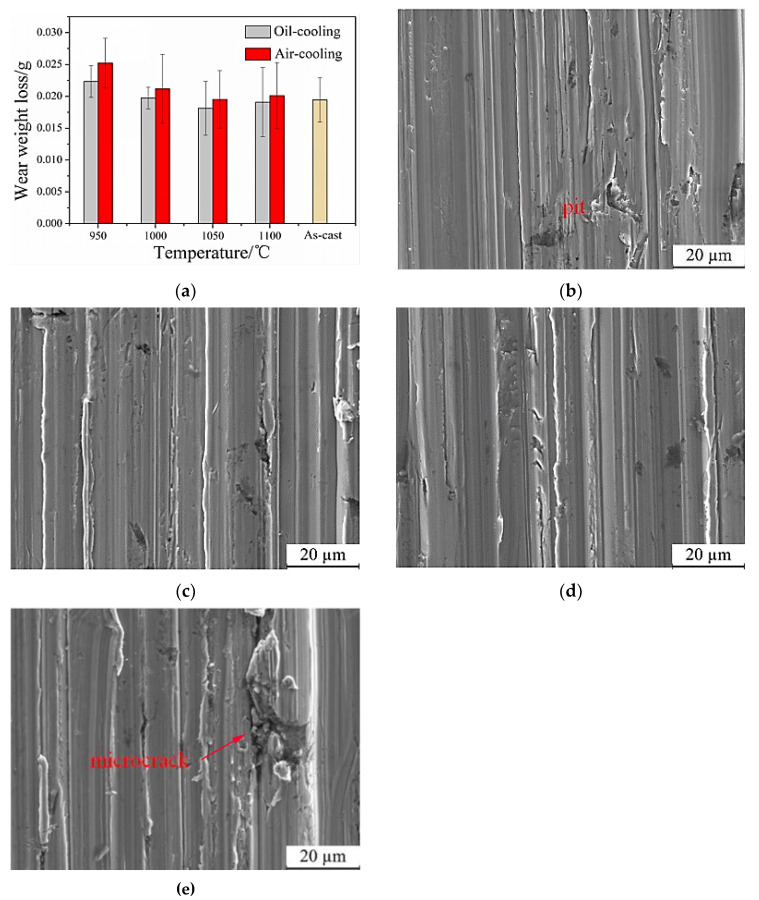
Wear weight loss and worn morphologies of low-carbon medium-chromium steel at various quenching temperatures. (**a**) Wear weight loss, (**b**) 950 °C, (**c**) 1000 °C, (**d**) 1050 °C, (**e**) 1100 °C.

Figure 17a displays the results of the abrasive wear test conducted on samples subjected to two different quenching media, oil and air, at various temperatures. The wear weight loss of the samples is recorded to evaluate the wear behavior. The figure demonstrates that the wear of the steel initially decreases and then increases with an increase in the quenching temperature. The minimum wear value is observed at a quenching temperature of 1050 °C. It is noteworthy that the wear resistance is influenced by the quenching temperature. Comparing the oil-quenched samples to the air-cooled samples at the same quenching temperature, it is evident that the oil-quenched samples exhibit lower wear. This suggests that quenching in oil results in better wear resistance compared to air cooling. Moreover, the wear resistance of the steel samples after heat treatment is not significantly different from those in the as-cast state. This indicates that heat treatment does not adversely affect the abrasive wear resistance of the steel. In other words, the steel maintains its wear resistance properties after undergoing the heat treatment process. Considering the overall results, the steel exhibits the best wear resistance at a quenching temperature of 1050 °C.

The worn morphologies of the steel, which have been annealed at 930 °C, then oil-quenched at various temperatures (950 °C, 1000 °C, 1050 °C, and 1100 °C), and finally tempered at 250 °C, are observed in Figure 17b–e. The figure illustrates that the wear morphologies of the steel are quite similar regardless of the quenching temperature. Micro-cutting and furrowing parallel to the wear direction are observed in all samples. This similarity can be explained by the presence of martensite in the microstructure after oil quenching at different temperatures. In particular, the sample quenched at 950 °C exhibits black pits on the surface, which can be attributed to the high hardness of the martensite. However, the residual austenite with relatively lower hardness, making it easier to wear off during the test. The wear surfaces of samples quenched at 1000 °C and 1050 °C appear uniformly flat and smooth, with shallow furrow depths due to the high hardness of these samples. On the other hand, cracks can be seen on the wear surface of samples quenched at 1100 °C, indicating a deeper furrow depth. Additionally, the surface shows signs of damage and contains numerous debris, possibly due to the high quenching temperature, which results in an increased amount of residual austenite in the microstructure. In this case, the presence of nano residual austenite can decrease the hardness of the steel and consequently reduce its wear resistance.

## 4. Conclusions

(1) The microstructure of the low-carbon medium-chromium steel typically consists of martensite and residual austenite network when in its as-cast state. However, after annealing at 930 °C, the microstructure undergoes a transformation into martensite and pearlite. It should be noted that both the hardness and impact toughness of the steel decrease compared to the original as-cast sample.
(2) The quenching temperature, ranging from 950 °C to 1100 °C, has a significant influence on the microstructure of the steel, namely martensite and nano residual austenite. Regardless of the quenching temperature, the hardness of the steel remains above 45 HRC, while the impact toughness continuously improves as the quenching temperature increases. The primary failure mode is dimple fracture, while quasi-dissociation fracture occurs as a secondary mode.
(3) At low tempering temperatures, an increase in tempering temperature leads to a decrease in the steel’s hardness. Initially, an increase in tempering temperature results in improved impact toughness, followed by a subsequent decrease in impact toughness. However, at a certain tempering temperature, the impact toughness begins to increase once again. The microstructure obtained after annealing at 930 °C, oil quenching at 1050 °C, and tempering at 250 °C primarily consists of martensite and fine nano residual austenite. This combination of microstructures contributes to well-balanced properties in the steel, with a hardness of 51 HRC and impact toughness of 40 J/cm^2^, representing a nearly fourfold improvement compared to the original as-cast sample.
(4) The wear weight loss of the steel exhibits a non-linear trend with increasing quenching temperature. Initially, the wear weight loss decreases as the temperature increases, but eventually starts to increase. The minimum wear weight loss is observed at a quenching temperature of 1050 °C. Additionally, at the same temperature, the wear weight loss of the oil-quenched sample is lower than that of the air-cooled sample. The steel with the highest wear resistance is obtained through the process of annealing at 930 °C, oil quenching at 1050 °C, and tempering at 250 °C.

## Figures and Tables

**Table 1:** The designed chemical composition and measured composition of low-carbon medium-chromium steel.

Elements	C	Cr	Si	Mn	Ni	Mo	Fe
Designed	0.20–0.22	10.00–12.00	0.50–0.65	0.60–0.80	0.80~1.00	0.35~0.40	Bal.
Measured	0.21	11.00	0.55	0.70	0.90	0.36	Bal.

**Table 2:** The number of heat treatment samples.

Sample	Annealing Temperature/°C	Quenching Temperature/°C	Quenching Media	Tempering Temperature/°C
QT1	930	950	Oil	250
QT2	930	1000	Oil	250
QT3	930	1050	Oil	250
QT4	930	1100	Oil	250
QM1	930	1050	Water	250
QM2	930	1050	Oil	250
QM3	930	1050	Air	250
T0	930	1050	Oil	200
T1	930	1050	Oil	250
T2	930	1050	Oil	300
T3	930	1050	Oil	350
T4	930	1050	Oil	400

**Table 3:** Parameters of the pin-on-disk two body wear test.

Project	Parameters
Sample size/mm	Φ6 × 20
Test load/N	50
Test distance/m	6
Test speed/mm·r^−1^	4
Disk speed/r·min^−1^	6

**Table 4:** Mechanical properties of the annealed low-carbon medium-chromium steel.

Sample	Hardness/HRC	Impact Toughness/J·cm^−2^
As-cast state	50	8.1
Annealing at 930 °C	41	6.4

## Data Availability

The data used to support the findings of this study are available from the corresponding author upon request.
